# The large X‐effect on secondary sexual characters and the genetics of variation in sex comb tooth number in *Drosophila subobscura*

**DOI:** 10.1002/ece3.2634

**Published:** 2016-12-20

**Authors:** Briana E. Mittleman, Brenda Manzano‐Winkler, Julianne B. Hall, Katharine L. Korunes, Mohamed A. F. Noor

**Affiliations:** ^1^ Biology DepartmentDuke UniversityDurhamNCUSA

**Keywords:** large X‐effect, secondary sexual traits, sex combs, sexual selection

## Abstract

Genetic studies of secondary sexual traits provide insights into whether and how selection drove their divergence among populations, and these studies often focus on the fraction of variation attributable to genes on the X‐chromosome. However, such studies may sometimes misinterpret the amount of variation attributable to the X‐chromosome if using only simple reciprocal F_1_ crosses, or they may presume sexual selection has affected the observed phenotypic variation. We examined the genetics of a secondary sexual trait, male sex comb size, in *Drosophila subobscura*. This species bears unusually large sex combs for its species group, and therefore, this trait may be a good candidate for having been affected by natural or sexual selection. We observed significant heritable variation in number of teeth of the distal sex comb across strains. While reciprocal F_1_ crosses seemed to implicate a disproportionate X‐chromosome effect, further examination in the F_2_ progeny showed that transgressive autosomal effects inflated the estimate of variation associated with the X‐chromosome in the F_1_. Instead, the X‐chromosome appears to confer the smallest contribution of all major chromosomes to the observed phenotypic variation. Further, we failed to detect effects on copulation latency or duration associated with the observed phenotypic variation. Overall, this study presents an examination of the genetics underlying segregating phenotypic variation within species and illustrates two common pitfalls associated with some past studies of the genetic basis of secondary sexual traits.

## 1 Introduction

Since Darwin's (1871) elegant elaboration, many studies have suggested that sexual selection is a powerful evolutionary force driving differences among individuals, populations, and species. One presumed outcome of sexual selection is sexual dimorphism and/or the evolution of secondary sexual traits, but such secondary sexual traits can also arise via natural selection or other processes. Genetic studies of traits can help elucidate whether they spread via selection (e.g., Orr, 1998) and which types of selection are most likely to have been involved (e.g., runaway sexual selection vs. good genes sexual selection; see Kirkpatrick & Hall, 2004). However, results thus far have sometimes contradicted theoretical expectations (Chenoweth & McGuigan, 2010). For example, while fitness‐related traits (sexually selected or otherwise) should generally retain little additive genetic variation within populations or species, various studies have shown that they often bear abundant heritable variation (e.g., Prokuda & Roff, 2014). Understanding the genetic underpinnings of fitness‐related traits, and how they interact with the environment, may help determine why genetic variation persists (Lynch & Walsh, 1998; Wilkinson et al., 2015).

Many studies have explored the X‐ (or Z‐)chromosome linkage of secondary sexual traits and behaviors in part because early work suggested that such sex‐chromosome linkage may be associated with adaptations (e.g., Ewing, 1969). Building on Haldane's (1924) classic observation that selection is unlikely to spread advantageous autosomal recessive alleles, Charlesworth, Coyne, and Barton (1987) showed that partially recessive favorable mutations were more likely to spread if on the X‐chromosome than if autosomal. They also found that this result held for male‐specific adaptations (like secondary sexual traits). Rice (1984) also showed that traits favored in one sex but unfavorable in the other would also be more likely to spread if controlled by genes on the X‐chromosome. As such, disproportionate X‐linkage may signal that a trait was adaptive. However, genetic studies of secondary sexual traits or genes involved in reproduction had contradictory results (see Chenoweth & McGuigan, 2010 for review). For instance, Reinhold (1998) reviewed the inheritance of 42 putatively sexually selected traits in reciprocal F_1_ crosses and found overall greater contributions of the X‐chromosome to this variation than to a sample of nonsexually selected traits. In contrast, Fitzpatrick (2004) studied the locations of 63 genes affecting putatively sexually selected traits and found that X‐linkage was not overrepresented.

While both of the above studies provide valuable insights into the genetics of secondary sexual traits, many such studies potentially oversimplify genetic analyses and use of the label “sexually selected.” Genetic studies of secondary sexual characters have sometimes estimated the relative contribution of genes on the X‐chromosome to a trait by comparing the phenotypic difference between offspring of reciprocal F_1_ crosses relative to the phenotypic difference between parental strains (e.g., Carson & Lande, 1984). XY offspring of reciprocal F_1_ crosses differ only in their sex chromosomes (and maternal effects), and thus, this approach should indicate how much variation is attributable to the sex chromosomes in particular. Although this approach has been advocated in the literature (e.g., Boake et al., 2002), it can overestimate the fraction of variation attributable to the sex chromosome because transgressive autosomal effects, as well as various epistatic effects, are not considered as part of the total genetic variance. This problem illustrates why researchers should use go beyond reciprocal F_1_ crosses to assess the X‐linkage of such variation. Additionally, although secondary sexual traits are sex‐specific, referring to such genes or traits generally as “sexually selected” is inappropriate without direct evidence. Further, even if a trait is sexually selected, specific variation in such traits or the genes affecting them may confer fitness differences, while other variation within the trait or gene may not. Finally, particular traits may be affected by sexual selection in some species groups but not others.

In this study, we sought to examine the genetic basis of segregating variation in a secondary sexual trait that has been shown to affect mating success in some systems: the Drosophila sex comb. Drosophila sex combs evolve rapidly between related species (see Kopp, 2011 for review), sex comb size sometimes correlates with mating success in wild flies (Markow, Bustoz, & Pitnick, 1996), and eliminating sex combs reduces male mating success (Cook, 1977; Hurtado‐Gonzales, Gallaher, Warner, & Polak, 2015; Ng & Kopp, 2008). However, quantitative manipulations in sex comb tooth number, similar in magnitude to differences observed between some Drosophila species, do not always affect mating success (Hurtado‐Gonzales et al., 2015). We focus on the sex combs of *Drosophila subobscura* because they are larger than in many other Drosophila species and known to exhibit segregating variation within and between natural populations (Beckenbach & Prevosti, 1986; Prevosti, 1955). Our objectives are to characterize the genetic basis of variation in this trait, to determine how well an estimate of X‐chromosome variation from reciprocal F_1_ crosses alone match variation inferred from a genotyped panel of F_2_ individuals (including specifically testing for transgressive effects), and to preliminarily assess whether the phenotypic variation affects copulation latency or duration within species.

## 2 Materials and Methods

### 2.1 Drosophila lines

We used 5 *D. subobscura* lines in this study: San Diego Stock Center ID 14011‐0131‐13 collected in the United Kingdom in 2008 (hereafter, “UK”); ID 14011‐0131.12 collected in Portugal in 2008 (hereafter, “Portugal”); ID 14011‐0131.05 collected in Germany in 2005; Seattle 6 collected in Seattle, Washington, USA, by Prof. Raymond Huey of the University of Washington in 2011; and Mount St. Helena (MSH), California, USA, 12 collected by Alexander Hish in 2013.

### 2.2 Sex comb tooth number phenotyping

*Drosophila subobscura* males have two sex combs (one proximal and one distal) on each of their front legs. We counted the individual teeth on each sex comb on each fly leg. Each leg was removed from the body with a scalpel and placed on a microscope slide covered with noble agar to hold it in place. For some samples, one leg was lost, but we used the remaining leg in measurements. We took digital photos of the slides on an Axioplan microscope with 100× amplification. For male progeny from the F_2_ cross, the remainder of the body was frozen for later DNA extraction. The average of the distal and proximal sex comb teeth number of the two legs from each fly was used for analysis when both legs were available (some were lost or damaged during dissection). This approach is justified in part because Nuzhdin and Reiwitch (2000) saw no consistent difference between left and right legs in sex comb tooth number. Further, we observed a strong correlation between the left and right distal sex comb tooth numbers among the F_2_ progeny surveyed (*N* = 596, *r* = .51, *p* < .0001), suggesting a shared component to their inheritance. Statistical significance of variation among inbred strains in proximal or distal sex comb tooth number was tested via a Kruskal–Wallis Test as implemented in R version 2.12.2 (Team, 2011).

To confirm the variation in distal sex comb tooth number between the UK and Portugal strains was not a byproduct of inbreeding, we performed an outcross of females from each of those strains to males of the Seattle 6 *D. subobscura* line. We measured the phenotype for 99 F_1_ progeny from the UK maternal cross and 102 F_1_ progeny from the Portugal maternal cross and used a Mann–Whitney *U* test in R to test whether the observed difference between the UK and Portugal was maintained in the outcrossed progeny.

### 2.3 Sequencing and marker development

No published genome sequence of *D. subobscura* or its close relatives exist, so we developed markers de novo. Total genomic DNA was phenol‐chloroform extracted from females of both pure strains (UK and Portugal) and sent to the Beijing Genomic Institute (BGI) for whole genome sequencing via Illumina HiSeq 2000, producing paired 150‐bp reads with inserts of 300 bp. Nardon et al. (2005) showed that *D. subobscura* genomes are similar in size to or smaller than *Drosophila melanogaster*. Assuming a conservative genome size of 175 Mb, the *D. subobscura* strains were each sequenced at ~60× coverage. The raw reads were filtered by BGI to remove adaptors, contamination, and low‐quality reads. Following these quality‐control steps, we received 68,935,864 reads for the UK strain and 69,247,710 reads for the Portugal strain.

De novo assembly of the genome sequence reads was performed using ABySS version 1.3.4 (Simpson et al., 2009) for each strain separately. For each strain (Portugal and UK), the raw data were initially contained in eight fastq files, which were combined to yield two fastq files per population: one containing the forward reads and one containing the reverse reads. ABySS was run on the Duke Compute Cluster using the command: “/opt/apps/bin/abyss‐pe *k* = 64 name=subobscuraUK_k64 in=‘uk_all_R1.fq uk_all_R2.fq’ ‐j 80,” where the specified input was the pair of sequence files for either Portugal or UK. The assemblies were performed once with a kmer of *k* = 64 and once with *k* = 75. For both populations, the *k* = 64 assembly yielded the better assembly, with N50 = 12,997 and N50 = 13,738 for Portugal and UK, respectively. Raw sequence reads were deposited in the NCBI Short Read Archive under BioProject accession PRJNA345020.

Primers for microsatellite markers RNAD and Amyrel were obtained from Noor, Pascual, and Smith (2000). To create additional primers for microsatellite or indel markers, we extracted sequences from the assembled contigs of each fly. We started by obtaining gene sequences from the published *Drosophila pseudoobscura* genome (Richards et al., 2005) on flybase.org (dos Santos et al., 2015). We BLASTed this sequence to both the Portugal and UK *D. subobscura* strain sequences locally (Altschul et al., 1997). We aligned the contigs obtained from the BLAST using the clustal alignment tool from embl‐ebi (Li et al., 2015) and designed primers flanking observed indels. The primers are named by the gene to which they correspond in the *D. pseudoobscura* genome. A subset of markers were chosen randomly on the chromosome of interest, and other markers were chosen for proximity to known in situ probes (Segarra & Aguade, 1992) to confirm sampling across the span of the X‐chromosome. Markers and primer sequences used are in Table S1.

### 2.4 Mapping crosses

The UK and Portugal lines were crossed (reciprocally) after virgins had been confined for 5–7 days; the original parents were collected and genotyped to confirm they had the expected genotype. The first cross (F_1_) virgins were collected and held in isolation for 5 days before being crossed again for 5–7 days to produce an F_2_ generation. At the end of the mating period, F_1_ parents were collected, genotyped for confirmation, and F_1_ males were phenotyped. In the next generation, 1152 F_2_ male flies were collected: 576 from the UK maternal derived cross and 576 from the Portugal maternal derived cross for subsequent genotyping and phenotyping.

### 2.5 DNA extraction and genotyping

A solution with 63.5 μl squish buffer (10 mmol/L Tris‐HCL (pH 8.2), 1 mmol/L EDTA, 25 mmol/L NaCl) + 1.3 μl proteinase K (Gloor & Engels, 1992) was added to the flies previously collected in 96 well plates. We placed a Zirconium bead in each well, sealed it with a plastic plate lid, and shook the plate on a Retsch TissueLyser II for 45 s to pulverize the fly. The product was incubated in a thermal cycler set to 37°C for 30 min, 95°C for 2 min, and 4°C for 4 min to inactivate the proteinase K.

We used the following reagents in our PCRs: 5 μmol/L forward primer + M13, 0.5 μmol/L reverse primer, 0.1 μmol/L 700IRD or 800IRD‐labeled M13 tag, 1.5 mmol/L MgCl_2_, 1× buffer, 0.2 mmol/L dNTPs, and 1 U Taq polymerase in a 10‐μl reaction volume. The PCR program included an initial denaturing step at 94°C for 60 s, three touchdown cycles 94°C–58°C–72°C for 30 s each, followed by 31 main cycles 94°C–56°C–72°C for 30 s each. I visualized the products on a 5% polyacrylamide gel using a LiCor 4300 DNA analyzer.

### 2.6 Test for location of genetic effect

As a first test for an X‐chromosome effect on distal sex comb tooth number, we used a Mann–Whitney *U* test in R to compare the average number of teeth on the distal sex combs of reciprocal F_1_ males. We tested whether there was a maternal effect on sex comb tooth number by comparing the average teeth number on the distal sex combs of F_2_ progeny with respect to the maternal line from which they were derived using a Mann–Whitney *U* test in R.

To localize gene(s) conferring an X‐chromosome effect on distal sex comb tooth number in F_2_ flies, we first used the onemap (Margarido, Souza, & Garcia, 2007) package as implemented in R version 2.12.2 to create a genetic map (in Kosambi centiMorgans) of the 13 X‐chromosome markers in 906 F_2_ flies. Onemap implements four two‐point based algorithms for marker ordering: seriation, rapid chain delineation, recombination counting and ordering, and unidirectional growth (see Margarido et al., 2007). The ordering of markers we present was consistent across at least three of the algorithms (if not all four).

Using this linkage map, QTL mapping was performed in R/qtl version 1.39 (Broman, Wu, Sen, & Churchill, 2003). This software makes use of the hidden Markov model to estimate QTL genotype probabilities. One marker was surveyed per autosome in 906 F_2_ males, but 13 markers were used to more comprehensively survey the X‐chromosome. An additional marker on the E and O chromosomes (Muller's elements C and E, respectively) was surveyed 192 of the F_2_ males to determine whether the chromosomes were inherited as a block because of the inversion polymorphisms in this species (Krimbas, 1992). Localization of X‐chromosomal effects and assessment of autosomal effects were performed with *scanone* via maximum likelihood using a Haley–Knott (1992) regression. Genomewide LOD significance thresholds with α of 0.01 were calculated with 100,000 permutations. The software used a hidden Markov model to then calculate QTL genotype probabilities across the X‐chromosome with a step size of 1 cM, and a plot of these probabilities is what is presented in Figure 2. We tested for two‐way interactions among factors in a multiple‐QTL model using *addint*, but none were statistically significant (*p* > .05 for all pairs). Percent variance explained was calculated in a multiple‐QTL model with *fitqtl*, adjusting for the effect of all QTL detected (the primary marker surveyed on each autosome and the peak association on the X‐chromosome) underlying the phenotype. R code used for this mapping, along with the raw genotype data, has been deposited in the Dryad digital repository (doi:10.5061/dryad.sc833).

### 2.7 Effects of distal sex comb tooth number on copulation latency or duration

As a trait expressed only in males and one whose ablation decreases mating success, we sought to test whether segregating variation in sex comb tooth number affected either copulation latency or duration and therefore was potentially sexually selected. Pairings were observed between F_2_ males and virgin F_1_ females, both 7 days posteclosion, and individually isolated for at least 24 hr before mating studies were conducted. An F_2_ male was combined with an F_1_ female in a single vial and given roughly a cubic inch of space in which to move, so that interactions between the two flies were maximized. The exact time (HH:MM:SS) was recorded as soon as the flies were combined. Flies were observed, and a second time was recorded as soon as the male mounted the female, allowing the copulation latency time to be calculated. A third time was recorded as soon as the male dismounted the female, allowing the copulation duration to be calculated. Matings that lasted <1 min were disregarded, and observations continued until a longer mating occurred. Only pairings in which male courtship was observed within 1 hr (>90% of trials) were recorded. Following mating, the male sex combs were dissected, photographed, and teeth counted as above. We tested for associations between sex comb tooth number (average of the two legs, larger of the two legs, smaller of the two legs, and difference between legs in sex comb tooth number) and either copulation latency or copulation duration via linear regression in R. We also tested for associations of average sex comb tooth number and either copulation latency or duration using a Cox proportional hazards regression (Andersen & Gill, 1982) in R using the *coxph* function within the package *survival*, and the results were unchanged relative to the linear regressions.

## 3 Results

### 3.1 Distal, not proximal, sex comb tooth number varies significantly across *D. subobscura* strains

**Figure 1:**
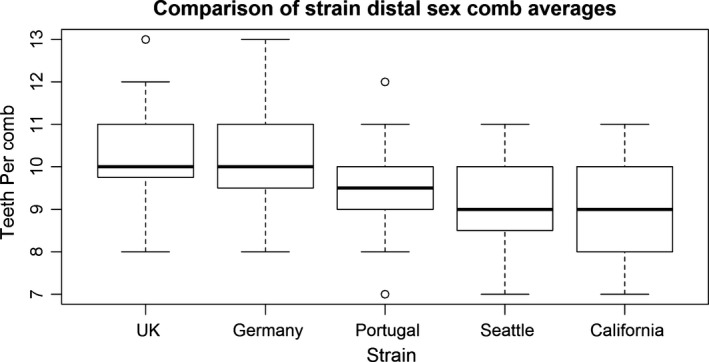
Boxplots of distal sex comb tooth numbers per leg across *Drosophila subobscura* strains

Proximal sex comb tooth number did not vary significantly among the five inbred strains surveyed (Kruskal–Wallis test, *p* = .41), whereas distal sex comb tooth number exhibited significant variation (Kruskal–Wallis test, *p* = 1.6 × 10^−6^; see Figure 1; Figure S1; Table S2). To test whether variation in teeth number on the distal sex combs was solely an artifact of inbreeding within the strains, we outcrossed the Portugal and UK lines (used for genetic mapping below) separately to the Seattle line. The difference in average number of teeth on the distal sex combs for the UK vs. Portugal lines was approximately as large as the difference in average number of teeth in the UK x Seattle vs. Portugal x Seattle crosses (0.8 vs. 0.9: the latter also showing a statistically significant difference between the UK and Portugal crosses—Mann–Whitney *U* test, *N* = 45 UK outcross/29 Portugal outcross, *p* = .000053, Table S1), demonstrating that differences observed in the homozygous lines are retained when made heterozygous.

### 3.2 Reciprocal F_1_ shows significant effect of X‐chromosome on distal sex comb tooth number

We observed statistically significant differences in the distal sex comb size between reciprocal F_1_ cross males (mean X^Portugal^: 10.29 teeth, mean X^UK^: 10.75 teeth, *N* = 100 X^Portugal^/105 X^UK^, Mann–Whitney *U* test, *p* = .0040, see Table S2). F_1_ males differ only in their sex chromosomes and maternal effects, and the direction of difference between the reciprocal F_1_ males matches the X‐chromosome/maternal origin. We used both reciprocal F_1_ crosses in the construction of the F_2_ generation, thus allowing us to determine whether maternal (e.g., cytoplasmic) effects drive the reciprocal F_1_ difference. However, the average distal sex comb tooth number in F_2_ flies with UK vs. Portugal maternal origin did not differ significantly despite the large sample sizes tested (*N* = 545 Portugal/552 UK, Mann–Whitney *U* test, *p* = .40, see Table S2), suggesting that one or more factors on the X‐chromosome rather than maternal effects contribute to the difference in distal sex comb tooth number between the UK and Portugal strains. The average phenotypic difference between reciprocal F_1_ males (0.46 teeth) was 57% as large as the difference between the parental strains.

### 3.3 Variation on all chromosomes correlates with distal sex comb tooth number in F_2_ progeny

Table 1 shows that the markers surveyed on each autosome exhibited significant associations with the variation in distal sex comb tooth number. The largest QTL (on chromosome I/J) had an effect opposite in direction to the phenotypic difference observed between the parental strains, illustrating the potential for transgressive segregation. We surveyed an additional marker on chromosomes E and O for a subset of the F_2_ males, and neither showed complete linkage to the primary marker tested (data not shown), demonstrating that recombination was occurring on these autosomes, and therefore, this study may be missing autosomal effects.

**Table 1:** Effect sizes on distal sex comb tooth number detected associated with markers in F_2_ progeny. Columns indicate the recombinational map position along the chromosome, chromosome, LOD score, *p*‐value, average homozygous/hemizygous Portugal‐strain phenotype, average homozygous/hemizygous UK‐strain phenotype, additive effect in joint model, and dominance effect in joint model

Position	Chrom	LOD	*p*	Port/Port	UK/UK	add	dom
199	A (X)	4.77	.00010	10.04	10.39	−0.206	NA
NA	E	4.21	.00038	9.80	10.31	−0.287	0.206
NA	I/J	9.45	<.00001	10.45	9.77	0.348	0.116
NA	O	8.13	<.00001	9.89	10.54	−0.302	0.025
NA	U	4.25	.00034	9.95	10.46	−0.254	−0.014

**Figure 2:**
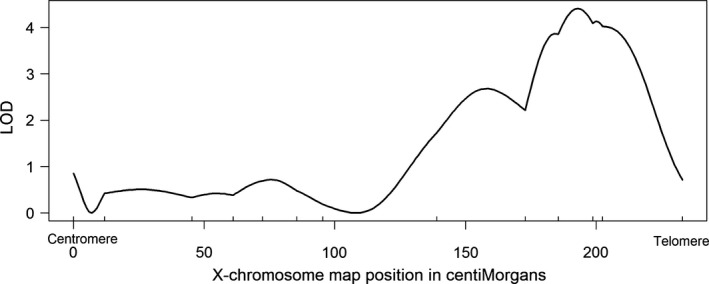
Interval map of distal sex comb tooth number variation association with positions along the X‐chromosome

Because of our interest in the X‐chromosome effect(s) in particular, we surveyed the X‐chromosome in far greater detail, using 13 markers rather than 1–2. Our QTL analysis shows evidence for a single significant LOD peak on the X‐chromosome correlating with distal sex comb tooth number among F_2_ flies (Figure 2). The effect is localized at position 199 cM in our linkage map and has a LOD score of 4.77 (*p* = .00038). The phenotypic difference between alternate genotypes at this locus among the F_2_ progeny (10.04–10.39: see Table 1) is ~77% the magnitude of the phenotypic difference between reciprocal F_1_ males, and therefore, this locus is likely the major contributor to the X‐chromosome effect detected in those F_1_ males. However, although this QTL provides further evidence for a significant X‐chromosome effect related to variation in distal sex comb tooth number, the phenotypic effect size is not disproportionate to QTLs on the autosomes: In fact, it has the smallest phenotypic difference observed between alternative homozygous/hemizygous classes (Table 1).

We constructed a joint QTL model using the four autosomal effects and the mapped X‐chromosome LOD peak (Table 1). This model, including one marker on each chromosome, explained 12.7% of the phenotypic variation. The estimated additive effect of the fine‐mapped X‐chromosome QTL was again the smallest observed among all the effects, suggesting that the X‐chromosome was not contributing a disproportionate effect.

### 3.4 No association detected between sex comb tooth number and copulation latency or duration

While our focus was to examine the genetic basis of this secondary sexual trait, we include here a small‐scale study testing whether the observed variation affects mating success, copulation latency, or copulation duration. We observed 393 pairings between outbred F_1_ females and courting F_2_ males. Of these, 365 pairings resulted in a copulation in the observation period. Of these pairings, we found no linear association between copulation latency or copulation duration and average distal sex comb tooth number, larger tooth number, smaller sex comb tooth number, or difference between legs in sex comb tooth number (*p* > .3 for all linear regressions; Figure S2 depicts associations for average distal sex comb tooth number). We also did not find any difference in average sex comb tooth between those which mated successfully and those which did not (10.11 vs. 10.12, *N* = 365/28, Mann–Whitney *U* test, *p* = .667).

## 4 Discussion

We identified hereditary variation among natural isolates of *D. subobscura* for a secondary sexual trait that affects mating success in some Drosophila species: sex combs. Using reciprocal F_1_ crosses, we observed what appeared to be a large effect of the X‐chromosome to distal sex comb tooth number differences between a strain from derived from the UK and one derived from Portugal. The phenotypic difference between reciprocal F_1_ males was 57% as large as the difference between the parental strains (equivalent to a 0.57 value of *I*_X_, as used by Reinhold (1998)) despite only ~20% of the genome being on the X‐chromosome. However, while this measure seemed to indicate a disproportionate X‐chromosome contribution to a sexually selected trait, further investigation showed that the QTL on the X‐chromosome had the smallest contribution of any of the chromosomes, and we did not detect evidence that phenotypic variation in this trait affected copulation latency, copulation duration, or probability of mating.

Disproportionate X‐linkage of traits may signal that they were adaptive, but our study highlights two common oversimplifications that have occurred in meta‐analyses and individual trait studies of the large X‐chromosome effect in secondary sexual traits. First, seemingly disproportionate effects of the X‐chromosome derived from reciprocal F_1_ crosses may be misleading. In this particular trait in these strains, the larger autosomal influence was masked because the largest effect QTL on an autosome had an effect opposite in direction to the difference between the parental strains. As such, the autosomal QTLs effectively canceled each other's effect in the F_1_, and their individual magnitudes could only be observed in the next generation. Similar opposing‐effect QTLs have been detected in sex comb mapping studies within *D. melanogaster* (Kopp, Graze, Xu, Carroll, & Nuzhdin, 2003; Nuzhdin & Reiwitch, 2000) or between Drosophila species (Macdonald & Goldstein, 1999; True, Liu, Stam, Zeng, & Laurie, 1997) as well (see Kopp, 2011 for review). While we did not detect any significant interactions among QTLs, epistasis can also complicate analyses from reciprocal F_1_ crosses alone and affect estimates of X‐chromosome effects.

Second, while studies often refer to secondary sexual traits as “sexually selected” based on findings that the trait is involved in mating or fertilization, specific variation in those traits need not be affected by sexual selection. Similar to a recent study in *D. melanogaster* and *Drosophila bipectinata* (Hurtado‐Gonzales et al., 2015), we failed to detect an effect of quantitative variation in *D. subobscura* sex comb tooth number on mating under these conditions. The variation studied in the F_2_ for such effects greatly exceeded the parental difference, with average tooth number ranging from 7.5 to 13, and yet we detected no hint of an effect on mating in our preliminary examination. Sex combs are thought to be used in tactile interactions between a courting Drosophila male and a female (Kopp, 2011), and complete ablation reduces mating success (Cook, 1977; Hurtado‐Gonzales et al., 2015; Ng & Kopp, 2008), so it was reasonable to assume effects on mating success, copulation latency, or copulation duration may be detectable with such wide variance in size even with a no‐choice pairing design.

Some studies had postulated that sex combs do not bear the same mating‐related function in the *melanogaster* group species as they do in the *obscura* group, wherein the former bear one sex comb row per front leg while the latter bear two rows (Cook, 1977; Kopp, 2011; Markow et al., 1996; Spieth, 1951). Cook (1977) noted that amputating the sex combs from *D. pseudoobscura* did not influence the male's ability to mate, but no numbers were given beyond “in the few operations performed successfully.” However, Spieth (1952) found that amputation of legs above the sex comb in *D. pseudoobscura* and *D. persimilis* dramatically reduced insemination rate relative to amputation below the sex comb, indicating a potential role of sex combs in mating success in the *obscura* group (see table 6 in Spieth, 1952).

Additional caveats also apply which are general to most forward‐genetic or behavioral studies of traits of interest. Foremost among these caveats is that such studies necessarily survey only a sample of the potential variation within the species. If a different pair of lines had been used, different results may have been obtained. Second, the lack of detected effect on mating may be because such effects are nonlinear, effects may be exclusive to particular conditions (such as on different food media: e.g., Etges et al., 2007), or testing under other different conditions (such as when given a choice of mates) may have changed the outcome. Finally, genetic or behavioral effects relevant on an evolutionary timescale may have been missed based on the necessarily limited scale of most laboratory studies.

Nonetheless, results of this study exemplify some of the dangers of extrapolation of the magnitude of X‐chromosome effects from reciprocal F_1_ cross data alone and assuming fitness effects of specific phenotypic variations in sexually selected structures. By extending to a subsequent generation, this work provides a first step to characterizing both the genetics and fitness effects of variation in a species bearing sex combs larger than most Drosophila species studied. Future work will characterize its genetic basis more fully and determine whether this extreme phenotype spread within *D. subobscura* by chance or via sexual selection. Presently, however, we cannot rule out that the size spread neutrally, and there is no indication of disproportionately large X‐linkage.

## Conflict of Interest

None declared.
