# Supplementary material for: The large X‐effect on secondary sexual characters and the genetics of variation in sex comb tooth number in Drosophila subobscura
Source: Ecol Evol. 2016 Dec 20;7(2):533–40. doi: 10.1002/ece3.2634 (PMC5243774; doi:10.1002/ece3.2634)

Supplementary Figure 1: Beeswarm plot overlaying boxplot from Figure 1 of distal sex comb tooth numbers per leg across *Drosophila subobscura* strains.


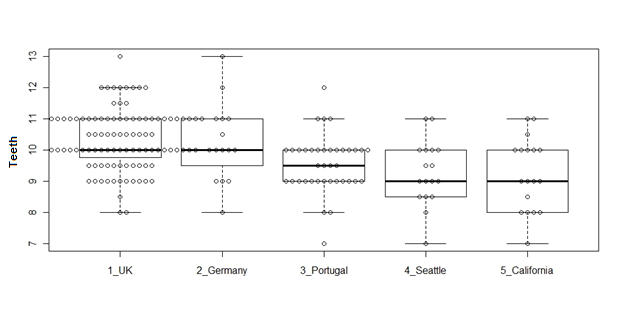

Supplement: Supplementary file 1 [file ECE3-7-533-s001.docx]
