# Supplementary material for: The large X‐effect on secondary sexual characters and the genetics of variation in sex comb tooth number in Drosophila subobscura
Source: Ecol Evol. 2016 Dec 20;7(2):533–40. doi: 10.1002/ece3.2634 (PMC5243774; doi:10.1002/ece3.2634)
Supplement: Supplementary file 2 [file ECE3-7-533-s002.docx]

Supplementary Figure 2: Association of copulation latency & duration with distal sex comb tooth number, both linear regressions and Cox proportional hazards regression


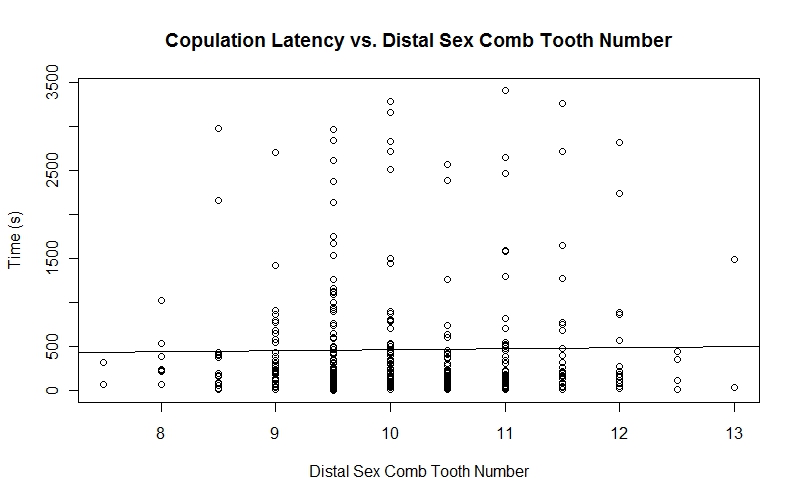
r^2^=0.00026, p=0.76


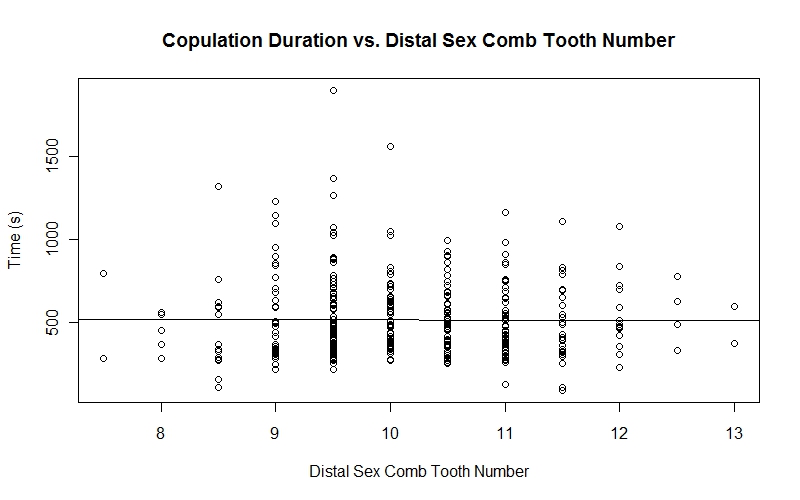


r^2^=1.7 x 10^-5^, p=0.94


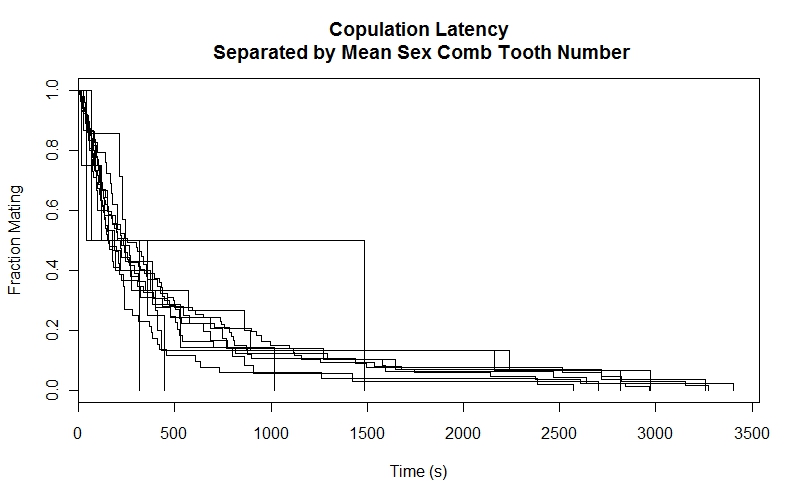


P=0.95


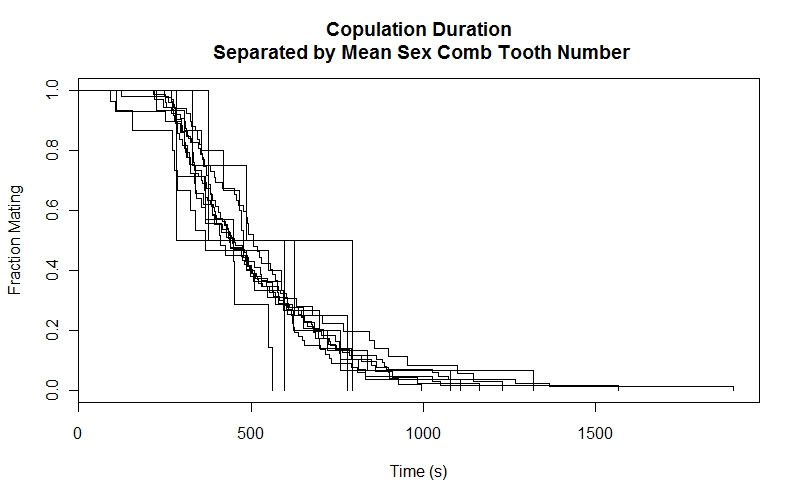


p=0.84
