# Supplementary material for: The large X‐effect on secondary sexual characters and the genetics of variation in sex comb tooth number in Drosophila subobscura
Source: Ecol Evol. 2016 Dec 20;7(2):533–40. doi: 10.1002/ece3.2634 (PMC5243774; doi:10.1002/ece3.2634)
Supplement: Supplementary file 4 [file ECE3-7-533-s004.docx]

Supplementary Table 2: Comparisons across strains and genotypes in average number of sex comb teeth per leg

Strain/ Genotype Sample size tested Average number teeth per leg

STRAINS: PROXIMAL SEX COMB:

Portugal 102 11.01

UK 99 10.75

Germany 15 11.03

California 18 10.44

Seattle 17 10.76

STRAINS: DISTAL SEX COMB

Portugal 102 9.52

UK 99 10.33

Germany 15 10.23

California (MSH) 18 9.22

Washington (Seattle) 17 9.26

OUTCROSSES TO MALES FROM SEATTLE STRAIN: DISTAL SEX COMB

Portugal F_1_ 29 9.74

UK F_1_ 45 10.60

RECIPROCAL F_1_ CROSS: DISTAL SEX COMB

Portugal mother F_1_ 100 10.29

UK mother F_1_ 105 10.75

RECIPROCAL F_2_ CROSS: DISTAL SEX COMB

Portugal grandmother F_2_ 545 10.27

UK grandmother F_2_ 552 10.19
